# The functional graphene/epoxy resin composites prepared by novel two-phase extraction towards enhancing mechanical properties and thermal stability

**DOI:** 10.3389/fchem.2024.1433727

**Published:** 2024-08-02

**Authors:** Wenfeng Zhu, Qianxi Wang, Pengchao Zhang, Lei Li, Li Zhang, Houbu Li, Lekang Ding, Zunlong Jin, Peipei Li, Jiaoxia Zhang

**Affiliations:** ^1^ Tubular Goods Research Institute, China National Petroleum Corporation and State Key Laboratory of Oil and Gas Equipment, Xi’an, Shaanxi, China; ^2^ School of Materials Science and Engineering, Jiangsu University of Science and Technology, Zhenjiang, China; ^3^ School of Mechanical and Power Engineering, Zhengzhou University, Zhengzhou, Henan, China; ^4^ School of Advanced Materials and Nanotechnology, Xidian University, Xi’an, Shaanxi, China; ^5^ School of Materials and Chemical Engineering, Ningbo University of Technology, Ningbo, China

**Keywords:** functionalized graphenes, two-phase extraction, epoxy resin, composites, mechanical properties and thermal stability

## Abstract

Epoxy resins, known for their excellent properties, are widely used thermosetting resins, but their tendency towards brittle fracture limits their applications. This study addresses this issue by preparing graphene oxide via the Hummer method, modifying it with hyperbranched polyamide ester, and reducing it with hydrazine hydrate to obtain functionalized graphene. This functionalized graphene improves compatibility with epoxy resin. Using a novel two-phase extraction method, different ratios of functionalized graphene/epoxy composites were prepared and tested for mechanical properties and thermal stability. The results showed significant improvements: the tensile strength of composites with 0.1 wt% functionalized graphene increased by 77% over pure epoxy resin, flexural strength by 56%, and glass transition temperature by 50°C. These enhancements, attributed to the improved compatibility between graphene and epoxy resin, demonstrate the potential of functionalized graphene to mitigate the brittleness of epoxy resins, expanding their application potential.

## Graphical Abstract

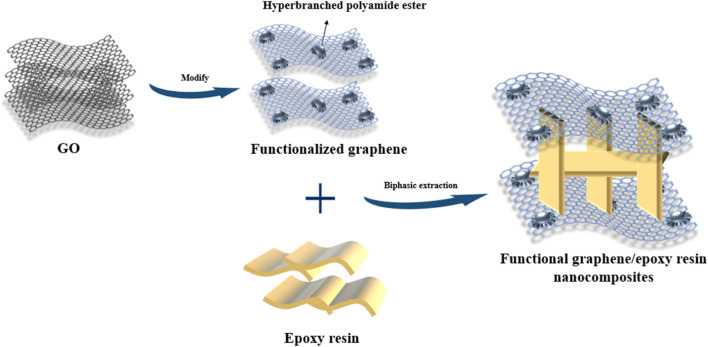

## 1 Introduction

Epoxy resin contains two or more epoxy groups in its molecule. Due to the chemical reactivity of the epoxy group, it can be cured and cross-linked by various compounds containing active hydrogen to form a three-dimensional network polymer (Kunrong et al., 2023). These network polymers have good mechanical properties, adhesion, corrosion resistance, high-temperature resistance, excellent electrical insulation, etc., due to the presence of multiple hydroxyl groups, aromatic groups, carbon chains, etc., and are widely used in aerospace, aerospace, electronics, automotive, construction and other fields (Yao et al., 2018). However, due to the structure of epoxy resin itself and the high crosslinking density after curing, it is prone to brittle fracture when it is subjected to external forces, and its toughness and impact resistance are poor, which restricts the development of epoxy resin (Duraibabu et al., 2022). Therefore, it is necessary to toughen epoxy resin, for example, by introducing rubber elastomers (Yahyaie et al., 2013; Yao et al., 2022), thermoplastic plastics (Kim et al., 2019; Yao et al., 2022), and nanomaterials (Li et al., 2016; Yao et al., 2018) to improve the toughness of epoxy resin.

Nanomaterials have different properties from bulk materials due to their large specific surface area, quantum size effect, macroscopic quantum tunneling effect, etc., and have triggered a research boom. When nanomaterials are introduced as a second phase into epoxy resin, it is found that they can improve the toughness of epoxy resin to a certain extent. In recent years, carbon nanomaterials (such as carbon nanotubes (Ming et al., 2022), and graphene (Zhang et al., 2019; Sahu and Raichur, 2022; Upadhyay et al., 2022) have been widely reported to effectively hinder the propagation of microcracks, thereby improving the strength and toughness of polymer matrix. Graphene is one of the thinnest and strongest materials ever discovered by humans, and thus becomes a potential material for engineering structures (Sharma et al., 2022). Graphene, which has a layered structure, especially oxidized graphene, has epoxy and hydroxyl functional groups on its surface due to oxidation, and is considered to be an ideal toughening material for epoxy resin (Chen et al., 2023). Functional graphene/epoxy composites have been widely applied in various fields such as aerospace, electronics, and medical industries. They are utilized for manufacturing structural materials, conductive materials, thermal management materials, anticorrosive coatings, biomedical devices, as well as energy storage and conversion equipment. By improving the mechanical properties and thermal properties of composites, not only can enhance their strength, stiffness, wear resistance, corrosion resistance, high temperature stability and flame retardancy, but also can achieve lightweight design, expand the scope of application, to meet the needs of high performance materials in various fields. The enhanced mechanical strength and stiffness allow for the creation of lighter structures without sacrificing durability, which can reduce the weight of vehicles and aircraft, improve fuel efficiency and performance. The increase in toughness means that the material can better withstand shock and fatigue, improving the life and reliability of components in various industries. Higher thermal conductivity helps to better dissipate heat, it can improve the reliability and longevity of electronic devices by maintaining lower operating temperatures.

However, nanomaterials also tend to agglomerate due to their large specific surface area, causing defects in the material or causing stress concentration, resulting in a decrease in the mechanical properties of the resin. Therefore, how to improve the interfacial strength between nanomaterials and epoxy resin matrix, and enhance the dispersion of nanomaterials in epoxy resin is a critical issue. Similarly, graphene, due to its large specific surface area and self-attraction within molecules, has a folded and wrinkled surface, which makes it easy to agglomerate in epoxy resin, resulting in a decrease in mechanical properties (Nayak et al., 2022). Therefore, surface modification is of great value for improving the compatibility of graphene and polymer matrix, and making the interface bonding between nanomaterials and matrix better. In addition, the addition of nanoparticles generally increases the viscosity of epoxy resin, further reducing the dispersion of nanoparticles in the matrix. Hyperbranched polymers, due to their compact structure, have lower viscosity to reduce the viscosity of the system, thereby improving the processability of the material (Misasi et al., 2017). Therefore, in this experiment, hyperbranched polyamide ester was first used to graft the surface of oxidized graphene with active groups, and then the modified oxidized graphene was further reduced to obtain functionalized graphene (Izadi et al., 2021). To further improve the dispersion of functionalized graphene in epoxy resin, functionalized graphene was first dissolved in water to form a graphene solution, then epoxy resin was added, and then a two-phase extraction method was used to transfer functionalized graphene from the aqueous phase to the epoxy resin system, and finally a curing agent was added to prepare functionalized graphene/epoxy resin composite. The bending and tensile tests exhibited the enhancement and toughening effects of functionalized graphene on epoxy resin, and the dynamic thermal mechanical properties showed the improvement of thermal properties (Zhang et al., 2019).

## 2 Experimental section

### 2.1 Materials

Hydrogen peroxide (AR, 37%), concentrated H_2_SO_4_ (AR, ≥98%), KMNO_4_, HCl from National Pharmaceutical Chemical Reagent Co, Ltd. Hydrazine hydrate (AR) from Shanghai Runjie Chemical Reagent Co., Ltd.; methyl tetrahydrophthalic anhydride (JHY910) from Jiaxing Lianxing Chemical New Material Co., Ltd.; epoxy resin E51, tris (dimethylaminomethyl) phenol (DMP30) from Wuxi Lanxing Resin Co., Ltd.; Hyperbranched polyamide ester and deionized water self-made.

### 2.2 Preparation of graphene

#### 2.2.1 Preparation of graphene oxide

The Hummers method is used to prepare graphene oxide (Izadi et al., 2021). Briefly, in a 500 mL beaker, 92 mL of H_2_SO_4_ and 2 g of NaNO_3_ were added into a beaker in a pre-prepared ice water bath and stirred slowly and evenly with a glass rod. 2 g of graphite powder was added into the solution and mixed evenly. Then, 6 g of potassium permanganate was slowly dropped for 5 min to allow the potassium permanganate to fully react in the ice water bath at all times. The reaction is complete after 1 h, the solution was standing for 24 h.

The standing solution was poured into a three-necked flask containing 92 mL of deionized water and stirred for 1.5 h at 35°C. Then, the temperature was adjusted to 95°C and reacted for 1 h. 6 mL of H_2_O_2_ was added into solution and stirred for 15 min. The solution is cooled to room temperature for filtration. The filter slag was dialyzed with dialysis bag for 3 days. The deionized water was replaced every 12 h. The suspension was centrifuged for 5 min at 5,000 rpm. The sediment is freeze-dried directly for 24 h to obtain graphene oxide.

#### 2.2.2 Functionalization of graphene

1 g of freeze-dried graphene oxide and 5 g of hyperbranched polyamide ester were dissolved in 150 mL of deionized water, to sonicate for 30 min to evenly disperse the graphene oxide in the water by a cell disruptor. Then, the solution was centrifuged for 5 min at 2000 rpm, and the upper turbidity was collected in a beaker. 0.78 g of hydrazine hydrate was added into the beaker to react for 24 h at 60°C by magnetic rotor. At last, the product was centrifuged for 5 min at 2000 rpm, and the upper turbidity was collected and freeze dry for 24 h to obtain functionalized graphene (GNHBPE).

### 2.3 Preparation of functionalized graphene/epoxy resin composites

An appropriate amount of functionalized graphene was dissolved in 25 mL of deionized water, then dispersed for 30 min using an ultrasonic crusher. After uniform dispersion, the vacuumed epoxy resin and functionalized graphene solution were poured into a 250 mL three-necked flask and heated up to 50°C under reflux conditions for 5 h under mechanical agitation. Then, the reflux device was removed and heated for 12 h at 48°C to remove the deionized water in the system. This results in a viscous resin, which is poured into a beaker. Further, curing agent and accelerator were added, poured the blending into a mold. Then the mold was placed into a vacuum drying oven and vacuumed to remove bubbles. Finally, the curing process was followed by 80°C/1 h + 120°C/3 h + 140°C/3 h to obtain the composites. Measure epoxy resin, methyl tetrahydro phthalic anhydride, and DMP30 in a mass ratio of 100:75:1 (He et al., 2024). The functionalized graphene is measured according to 0 wt%, 0.05 wt%, 0.1 wt%, 0.2 wt%, and 0.3 wt% of the mass of the epoxy resin. All samples are prepared using the same method.

### 2.4 Testing and characterization

The infrared spectrum of functionalized graphene and graphene oxide is measured using the American-made FTS2000 Fourier infrared spectrometer, using the potassium bromide tablet. The Invia Renishaw laser Raman spectrometer from Renishaw International Ltd. In the United Kingdom is used to test and analyze graphene oxide and functionalized graphene. The scanning range is 900-2000 cm^−1^. The Pyris Diamond TG-DTA/DSC from PerkinElmer is used to test the thermal stability of functionalized graphene with a heating rate of 15°C/min and a temperature range from room temperature to 600°C. Graphene obtained by the two-phase extraction method is evenly smeared on a glass slide, then the cover glass is covered, the bubbles are removed, and then the morphology of functionalized graphene is observed with a polarizing microscope at different magnifications.

The tensile and bending properties of prepared functionalized graphene/epoxy resin composites are executed by the universal material testing machine Criterion 40 from MTS according to GB/T 2567–2008, with sample sizes of 150 mm*10 mm*4 mm (dumbbell samples) and 80 mm*15 mm*4 mm (rectangular samples). Each test is done with five sets of samples to obtain the averaged values. The German NEZSCH 242C DMA machine is used, to perform dynamic mechanical scanning using a three-point bending mode with a temperature range from 20°C to 200°C, and a heating rate of 5°C/min at the 1 Hz of frequency, and the100 μm of amplitude. Finally, the functionalized graphene/epoxy resin nanocomposites and graphene oxide samples are fixed on the carrier with conductive glue, then gold is sprayed to make the material conductive, and then the tensile fracture morphology is observed at different magnifications by the SEM JSM-IT800 from JEOL.

## 3 Result and discussion

### 3.1 Structural analysis of functionalized graphene

**FIGURE 1:**
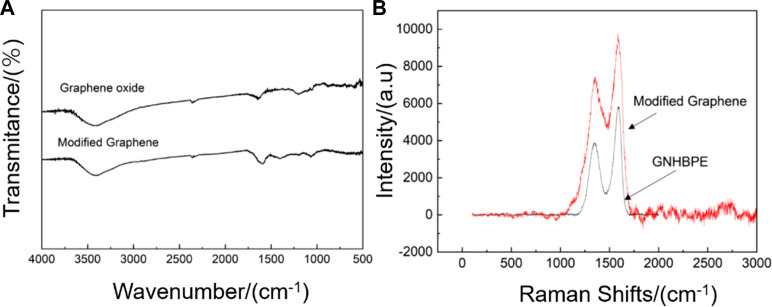
**(A)** Infrared spectra and **(B)** Raman spectra of hyperbranched polyamide ester-modified graphene and graphene oxide.

Figure 1A is the FTIR spectrum of graphene oxide and hyperbranched polyamide ester modified graphene. It can be found that a strong absorption peak appears in the graphene oxide at 3400 cm^−1^–3500 cm^−1^, which is due to the presence of a large amount of -OH, making graphene oxide have a strong absorption peak. The peak appearing at 1,500–1550 cm^−1^ is the vibrational contraction peak of the carboxyl group’s C-O, the peak appearing at 1550 cm^−1^–1600 cm^−1^ is the vibrational contraction peak of C = O, and the weak peak appearing near 900 cm^−1^ is the epoxy group peak, indicating that the oxidation degree of graphene oxide is good. The strong absorption peak appearing at 3400 cm^−1^–3500 cm^−1^ in the infrared spectrum of hyperbranched polyamide ester-modified graphene is the vibrational contraction peak of -OH and -NH, the peak appearing at 1,589 cm^−1^ is the -NH bending vibration peak in hyperbranched polyamide ester, and the peak appearing at 1,072 cm^−1^ is the C-N stretching vibration peak. This indicates that the modification of graphene by hyperbranched polyamide ester has been successful.

Figure 1B is the Raman spectrum of graphene oxide and hyperbranched polyamide ester-modified graphene. For graphene oxide, the G peak appearing around 1,600 cm^−1^ is the characteristic peak of the carbon sp2 structure, reflecting the symmetry and crystallinity of graphene oxide. The D peak appearing around 1,350 cm^−1^ is a defect peak, reflecting the disorder of the graphene layer, the disorder of the boundary edge area of general graphene, the intensity ratio of D peak and G peak (ID/IG) is used to represent the regularity of the graphite structure of carbon nanomaterials. The ID/IG of graphene oxide is 0.66, and the modified graphene by hyperbranched polyamide ester has peaks at both G peak and D peak relative to graphene oxide. The ID/IG value of functionalized graphene is 0.78, which is higher than that of graphene oxide, indicating that the regularity has decreased. This is because although graphene oxide has been reduced, the surface grafting of disordered hyperbranched polyamide ester has led to a decrease in its regularity (Zhang et al., 2024).

**FIGURE 2:**
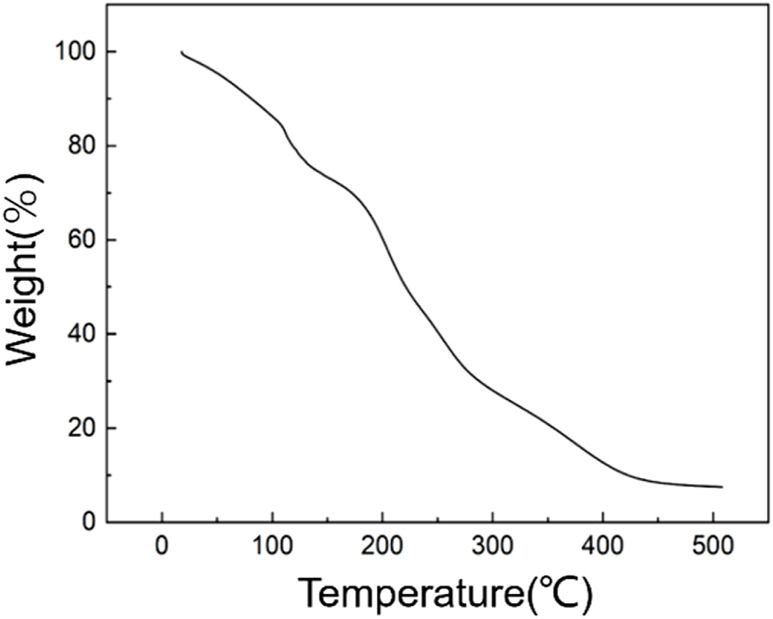
Thermogravimetric curves of functionalized graphene.

Thermogravimetric analysis can be used to study the thermal stability of the materials. Figure 2 shows the thermogravimetric curve of functionalized graphene. The mass of the functionalized graphene has already decreased by 20% at temperatures between 20°C and 120°C, proving that there are small amounts of solvent and water molecules in the graphene. The decrease in the mass of graphene is mainly from 120°C to 425°C. The mass decrease from 120°C to 250°C is mainly due to the loss of functional groups on the surface of the graphite and hyperbranched polyamide ester, with a mass reduction of more than 40%. The mass change between 250°C and 500°C is mainly due to the destruction of the main chain structure of the hyperbranched polyamide ester, leading to the failure of functionalization. After the temperature exceeds 500°C, the remaining mass is more than 10%, which is the content of the residual graphene.

**FIGURE 3:**
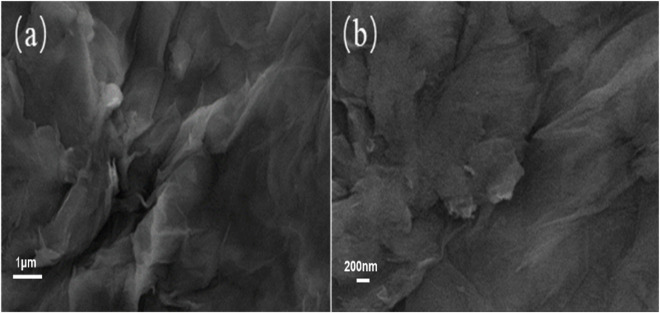
**(A)** SEM of graphene oxide and **(B)** functionalized graphene.

The morphology of graphene oxide and functional fossil ink is depicted in Figure 3, as observed through scanning electron microscopy. It is evident that the surface of oxidized graphene exhibits a relatively intact structure with no signs of fragmentation, displaying a wrinkled sheet structure. The modification and reduction reaction of hyperbranched polyamide ester resulted in a significant reduction in wrinkles for the functionalized graphene.

### 3.2 Study on the dispersion of functionalized graphene in water and epoxy resin

**FIGURE 4:**
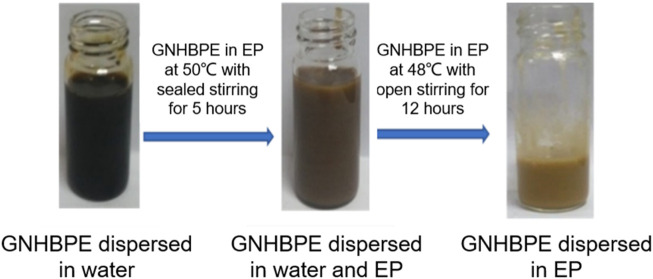
Dispersion photos of functionalized graphene at different periods.

Due to the large van der Waals forces between layers, graphene is difficult to separate from foreign substances and forces, making it difficult to disperse in polymers. To increase the compatibility of functionalized graphene in epoxy resin and prevent its agglomeration, we use a two-phase extraction method to treat graphene during the preparation of functionalized graphene/epoxy resin composites to prevent graphene from agglomerating in epoxy resin. Figure 4 shows the dispersion of functionalized graphene at different periods. The graphene is gradually transferred from the water to the epoxy resin, and their color is gradually lightened. Because the surface of graphene still contains considerable polar functional groups, it has good dispersion in water. In epoxy resin, due to the relatively large viscosity of epoxy resin, graphene is wrapped in epoxy resin, resulting in different refractive indices, resulting in lighter color. It can be seen that GNHBPE has good dispersion at different stages. When GNHBPE is dispersed in water, the amine, ester, and hydroxyl groups contained in the hyperbranched polyamide ester itself allow graphene to be evenly dispersed in water. Due to the amphiphilicity of the hyperbranched polyamide ester itself, stir the GNHBPE aqueous solution and epoxy resin with a high-speed agitator to fully mix them, without obvious layering and uneven parts, to form a uniform emulsion. When heating to 48°C to remove water from the system, the dispersed functionalized graphene gradually transfers from the water phase to the epoxy resin phase. This process is a phase transfer process from water to epoxy resin. As the water gradually evaporates, the dispersed functionalized graphene slowly migrates into the molecular network structure of the epoxy resin during the gradual evaporation of the water phase. In the whole preparation process, functionalized graphene shows well dispersion in both water and epoxy resin.

**FIGURE 5:**
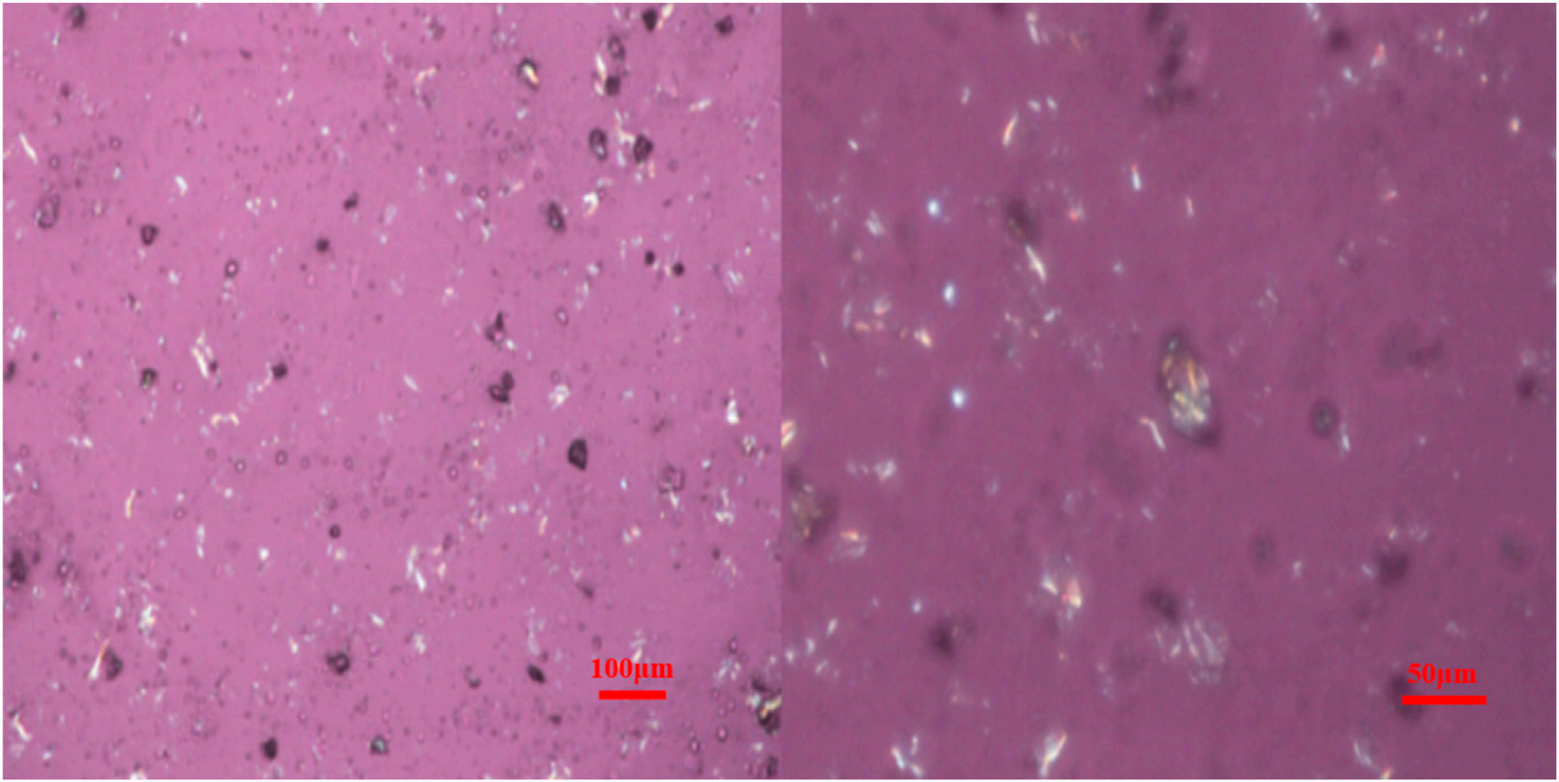
Polarized micrograph of functionalized graphene after two-phase extraction.

When observing the crystalline or orientation in polymers with a polarizing microscope, the optical properties vary with the direction of incident light. When the vibration direction of the crystal is inconsistent with the direction of the r vibrators, the structural morphology of the substance can be observed. As shown in Figure 5, the functionalized graphene after two-phase extraction was observed under a polarizing microscope, and it is not difficult to find that the functionalized graphene exhibits crystallization. Crystalline graphene is expected to enhance the toughness of composites and increase heat resistance.

### 3.3 Mechanical properties

**FIGURE 6:**
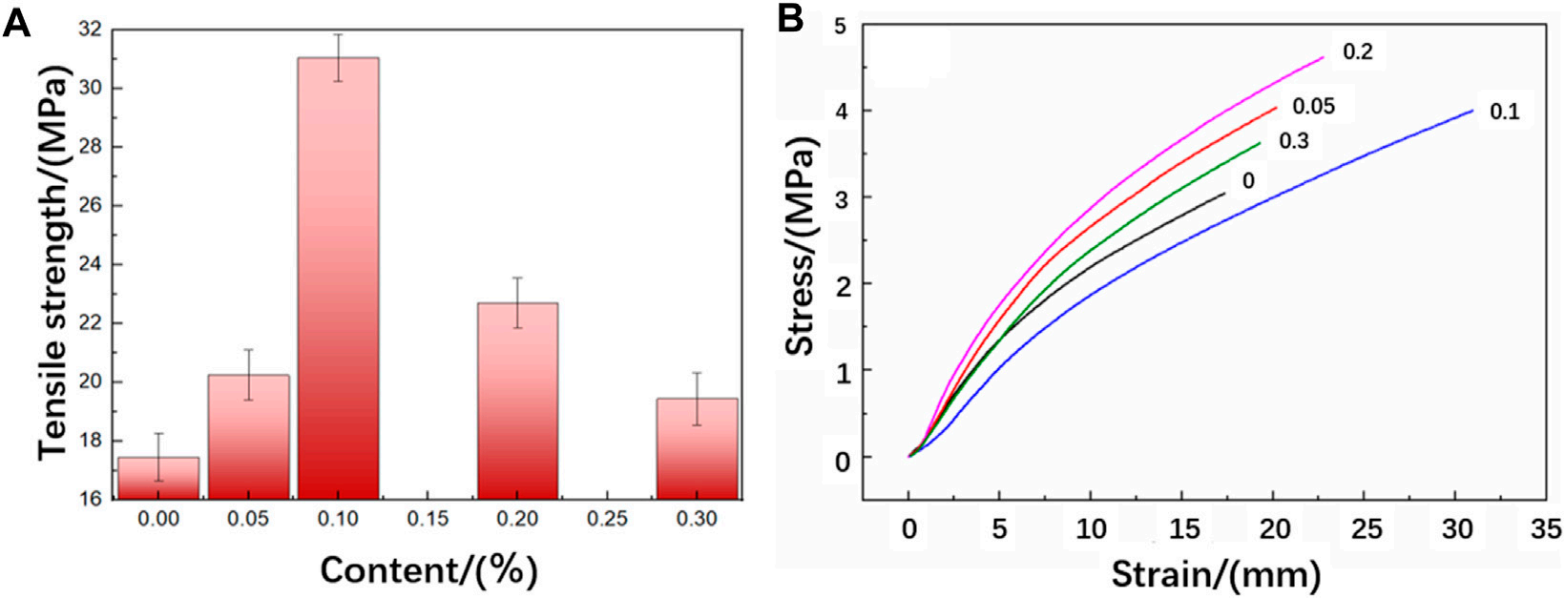
**(A)** Tensile strength of functionalized graphene/epoxy composites **(B)** Stress-strain curves of functionalized graphene/epoxy composites.

Table 1 shows the tensile properties of functionalized graphene/epoxy resin composites with different functionalized graphene contents. The tensile strength of pure epoxy resin is 17.46 MPa, and the tensile strength of functionalized graphene/epoxy resin with a mass fraction of 0.1 wt% is 31.02 MPa, which is 177% of the tensile strength of pure epoxy resin, indicating a significant increase in the strength of the composites. The fracture elongation of pure epoxy resin is 3.88%, and its fracture elongation increases first and then decreases with the increase of functionalized graphene content. The fracture elongation of the functionalized graphene/epoxy resin composites with 0.1 wt% is 5.32%, and the fracture elongation rate has increased by 37%. In addition, Figure 6B shows the stress-strain curve of the functionalized graphene/epoxy resin composites. It can also be seen that when the mass fraction of functionalized graphene is 0.1wt%, the strain increases under the same stress conditions, indicating that the toughness of the composites has increased.

**TABLE 1:** Tensile property data for pure epoxy sheets and different ratios of functionalized graphene sheets.

Quantity contained (%)	Elongation at break/%	Tensile strength/MPa	Tensile breaking stress/MPa	Yield stress/MPa
0	3.88	17.46	0.745	0.733
0.05	4.52	20.20	0.666	0.658
0.1	5.32	31.02	1.747	0.86
0.2	4.85	22.81	0.569	0.565
0.3	4.60	19.35	0.534	0.524

Figure 6A shows the tensile strength of the functionalized graphene/epoxy resin composites. It can be seen that the tensile strength first increases and then decreases with the increase of the content of functionalized graphene, but it increases to varying degrees compared to the pure epoxy resin sheet. When the content of functionalized graphene reaches 0.1 wt%, the tensile strength reaches its maximum. As the graphene increases, the possible reasons for the enhancement of the mechanical properties of the composites are as follows: Firstly, the excellent mechanical properties of graphene play an enhancing role in the composites. In addition, the amines contained in the hyperbranched polyamide ester grafted on the functionalized graphene will react with the curing agent, thereby further strengthening the interfacial adhesion strength of graphene and epoxy resin. When it is subject to external forces, it can reduce the debonding of graphene and epoxy resin, produce more micro-cracks, absorb more energy, and thus improve the tensile fracture strength. At the same time, when the content of functionalized graphene is 0.1 wt%, the fracture elongation rate of the composites also reaches the optimal value, which is 16% higher than that of pure epoxy resin. This is because both the hyperbranched polyamide ester and graphene themselves have good flexibility. In addition, the branched structure of the hyperbranched polyamide ester itself leads to more free volume in the system, thereby enhancing the mobility of the chain segment, which can absorb energy from all directions, and the force-induced base curve deformation makes the crack blunt, thereby effectively improving the toughness of the epoxy resin. However, when it exceeds 0.1 wt%, the tensile strength significantly decreases. This is because the purpose of modifying graphene is to increase the compatibility of graphene and epoxy resin. When it exceeds the critical value, the dispersibility of graphene in epoxy resin will decrease, and the agglomeration phenomenon will cause its mechanical properties to decrease.

Table 2 shows the bending properties of functionalized graphene/epoxy resin composites. The bending modulus of the pure epoxy resin without added functionalized graphene is 2,698.36 MPa, and the bending modulus of the composites with a functionalized graphene content of 0.1 wt% is 3,395.02 MPa, an increase of 25% from the former. In addition, the bending strength of the pure epoxy resin sheet is 91.87 MPa, and the bending strength of the composite sheet with a functionalized graphene content of 1% is 144.73 MPa, an increase of 56% from the former.

**TABLE 2:** Flexural properties of functionalized graphene/epoxy composites.

Samples with different ratios of functionalized graphene	Flexural modulus/MPa	Bending strength/MPa	Bending stress at specified deflection/N	Bending rupture stress/N
pure epoxy sheet	2,698.36	91.87	93.82	95.76
0.05%	2,950.85	105.34	105.40	105.78
0.1%	3,395.02	144.73	136.57	144.07
0.2%	2,912.68	99.87	103.57	105.55
0.3%	2,933.54	93.62	94.45	95.21

**FIGURE 7:**
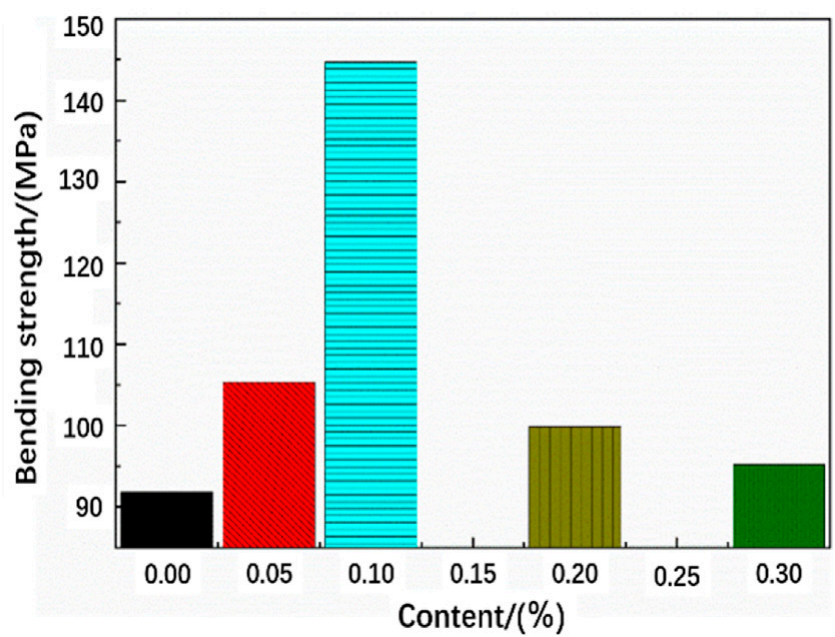
Flexural strength of functionalized graphene/epoxy composites.

Figure 7 shows the change of the bending strength of the functionalized graphene/epoxy resin composites. The bending strength and bending modulus of the composite increase with the increase of the functionalized graphene content. The bending strength and bending modulus reach their maximum values when the graphene content reaches 0.1 wt%, which is consistent with the previous tensile test results. At the same time, when the content exceeds 0.1 wt%, the bending strength and bending modulus gradually decrease. When the content of graphene is high, it will cause the dispersibility of graphene in epoxy resin to decrease, and it is easy to cause graphene agglomeration and defects during the production of composite sheets. These defects will generate stress concentration during the bending process, thereby reducing the bending strength and bending modulus.

**FIGURE 8:**
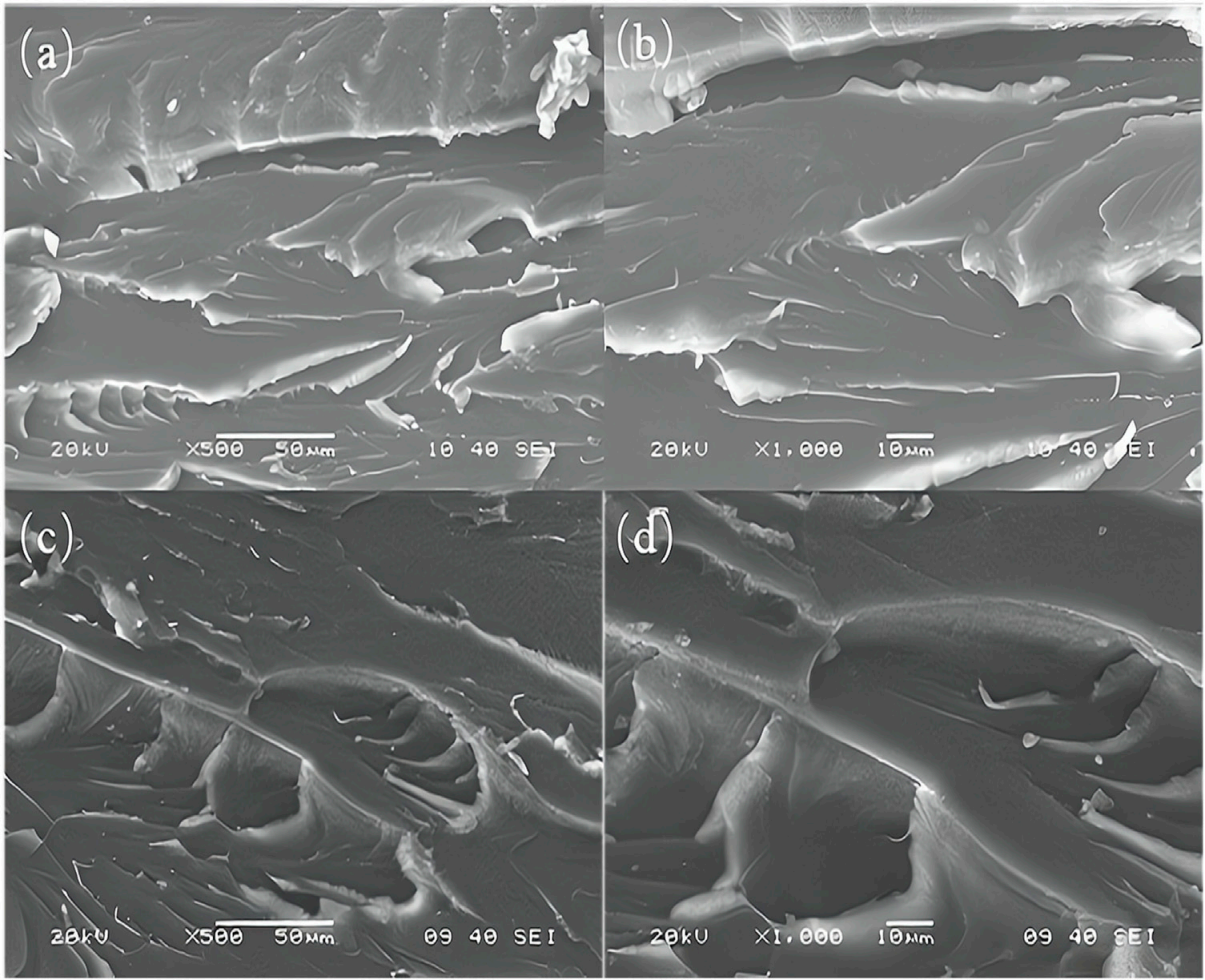
**(A)** and **(B)** are the cross-section morphologies of pure epoxy resin, and **(C)** and **(D)** are the cross-section morphologies of functionalized graphene/epoxy resin composites containing 0.1%.

Brittle fracture refers to the fracture that occurs before the material has plastic deformation, while ductile fracture refers to the fracture that occurs after the material has obvious macroscopic plastic deformation. Figure 8 shows the fracture morphology of pure epoxy resin and 0.1 wt% functionalized graphene/epoxy resin composites. It can be seen that the fracture of pure epoxy resin is relatively smooth, with obvious river-like structures, which conform to brittle fracture. For the composites with added functionalized graphene, the river-like surface of the fracture produces obvious cracks. The existence of these micro-cracks proves that the composites belong to ductile fracture, which can absorb the energy of external forces, thereby effectively protecting the matrix from damage, making it not easy to fracture, and thus increasing the toughness of the composites. Functionalized graphene improves interfacial adhesion by forming strong bonds with the resin matrix, thereby reducing crack initiation and propagation. The high aspect ratio and stiffness of graphene act as effective reinforcement, transferring stress within the composite and enhancing overall strength. Additionally, graphene’s barrier properties inhibit moisture ingress, preserving the composite’s integrity. Toughening mechanisms like crack deflection and bridging further increase fracture toughness and impact resistance. Uniform dispersion and potential synergies with other additives ensure consistent enhancement throughout the material, making functionalized graphene a versatile and effective modifier for epoxy resin composites.

### 3.4 Dynamic mechanical properties

Dynamic mechanical analysis is a study of the trend of the dynamic mechanical properties of polymers with temperature at a fixed frequency. Polymers have viscoelasticity, meaning they exhibit both viscosity and elasticity (Li et al., 2023). When studying dynamic mechanical properties, we usually use alternating stress to obtain a temperature spectrum. The temperature spectrum of dynamic mechanics can not only obtain characteristic temperatures such as Tg (glass transition temperature), Tf (freezing temperature), Tβ, Tγ, Tδ, etc., but also obtain damping and other properties, which are widely used in material research (Serra-Aguila et al., 2022).

**FIGURE 9:**
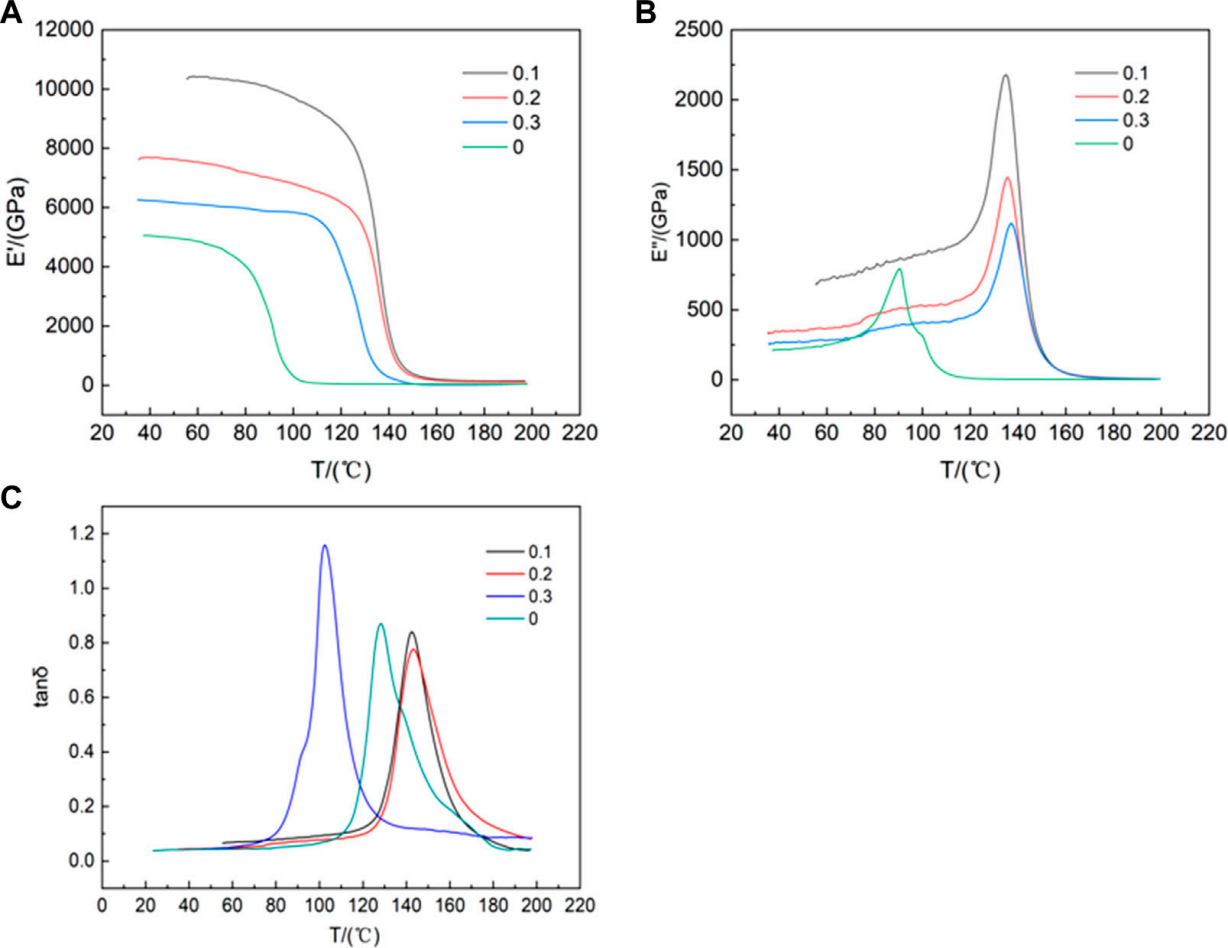
**(A)**Temperature dependence of storage modulus of functionalized graphene/epoxy composites at 1 Hz **(B)** 1 Hz Loss Modulus of Functionalized Graphene/Epoxy Composites as a Function of Temperature **(C)** 1 Hz Loss Factor Curve of Functionalized Graphene/Epoxy Composites with Temperature.

The storage modulus, also known as the elastic modulus, is essentially Young’s modulus, which is used to describe the ability of a material to store elastic deformation. Figure 9A shows the storage modulus of the functionalized graphene/epoxy resin composites at 1 Hz with temperature increasing. The storage modulus of the pure epoxy resin is large below 72°C, which is in the glassy state. In the glassy state, the chain segments of the polymer have not fully relaxed, and the main moving parts are chain joints, side groups, etc. Therefore, the storage modulus is relatively large. From 72°C to 105°C, the main moving unit is the chain segment, due to the slippage of the chain segment, the storage modulus decreases in the high elastic state. Above 105°C, the composites are in the viscous flow state, the movement is caused by the slip of the molecular chain, so the storage modulus is minimized. The composites with a mass fraction of 0.1wt% functionalized graphene are in the glassy state when it is below 120°C, in the high elastic state from 120°C to 145°C, and in the viscous flow state when it is above 145°C. Compared with pure epoxy resin, the functionalized graphene/epoxy resin composites have greatly improved their storage modulus and thermal stability.

This may be related to the degree of dispersion of graphene in epoxy resin. The better the dispersion of graphene in epoxy resin, the better the mechanical properties of the composites. The loss modulus is used to describe the phenomenon of energy being converted into heat when the material deforms. Figure 9B shows the curve of loss modulus of the functionalized graphene/epoxy resin composites at 1 Hz. As can be seen from Figure 9B, the glass transition temperature (Tg) of the epoxy resin is about 85°C, while the glass transition temperature Tg of the composites with a mass fraction of 0.1 wt% functionalized graphene is 135°C, which is 50°C higher than that of the pure epoxy resin. Tg is a temperature of amorphous polymers, at which the increase in molecular mobility leads to significant changes in thermal properties. Polymers with high Tg values have better heat resistance and are not easily degraded at high temperatures, which is crucial in determining the characteristics and properties of polymers. This shows that functionalized graphene greatly improves the thermal stability of the composites. This may be because functionalized graphene itself has higher thermal stability, so it enhances the thermal stability of the composites.

The loss factor δ) is numerically equal to the ratio of the loss modulus to the storage modulus, and is related to the material damping. In Figure 9C, for pure epoxy resin, when the temperature is lower than Tg, the material is in a glassy state, and the deformation is mainly caused by the movement of the chain joints, so δ is relatively small meaning the low damping. When the material transitions from a glassy state to a high elastic state, the deformation is mainly caused by the movement of the chain segments, so δ is relatively large with the increasing damping. As can be seen from Figure 9C, the peak of pure epoxy resin appears at about 128°C, while the peak of the composites with a mass fraction of 0.1 wt% functionalized graphene appears at about 143°C. The peak strengths of the two are not much different, but the width of the peak of the functionalized graphene/epoxy resin composite sheet with a mass fraction of 0.1 wt% is greater than that of the pure epoxy resin peak, so the damping is relatively increased.

## 4 Conclusion

The synthesized graphene oxide was modified using hyperbranched polyamide ester and reduced to yield functionalized graphene. The functionalized graphene/epoxy resin nanocomposites were subsequently fabricated utilizing a biphasic extraction technique. The biphase extraction method enhances the dispersion and compatibility of graphene in epoxy resin, enhances the adhesion between functional graphene and epoxy resin, reduces the interfacial shear stress, and thus improves its mechanical properties. Concurrently, with 0.1 wt% functionalized graphene, the bending strength and tensile strength of the epoxy resin composites are 144.73 MPa and 31.02 MPa, respectively. These values represent an increase of 56% and 77% compared to a pure epoxy resin system. The incorporation of functionalized graphene further elevates the thermal stability of the epoxy resin, with the glass transition temperature (Tg) reaching 143°C, a rise of 50°C from that of pure epoxy resin.

## Data Availability

The raw data supporting the conclusions of this article will be made available by the authors, without undue reservation.
